# Multi-Physics Coupling Modeling and Experimental Investigation of Vibration-Assisted Blisk Channel ECM

**DOI:** 10.3390/mi13010050

**Published:** 2021-12-29

**Authors:** Juchen Zhang, Shasha Song, Junsheng Zhang, Weijie Chang, Haidong Yang, Huohong Tang, Shunhua Chen

**Affiliations:** 1 School of Mechanical Engineering, Hefei University of Technology, Hefei 230009, China; 2020110218@mail.hfut.edu.cn (S.S.); zhangjunsheng90@126.com (J.Z.); changwj1981@163.com (W.C.); yanghaidonghfut@126.com (H.Y.); tanghh@ustc.edu.cn (H.T.); shchen@hfut.edu.cn (S.C.); 2 College of Mechanical and Electrical Engineering, Nanjing University of Aeronautics & Astronautics, Nanjing 210016, China

**Keywords:** vibration-assisted electrochemical machining (ECM), blisk, narrow channel, high aspect ratio, multi-physics coupling simulation, machining stability

## Abstract

Due to its advantages of good surface quality and not being affected by material hardness, electrochemical machining (ECM) is suitable for the machining of blisk, which is known for its hard-to-machine materials and complex shapes. However, because of the unstable processing and low machining quality, conventional linear feeding blisk ECM has difficulty in obtaining a complex structure. To settle this problem, the vibration-assisted ECM method is introduced to machine blisk channels in this paper. To analyze the influence of vibration on the process of ECM, a two-phase flow field model is established based on the RANS *k-ε* turbulence model, which is suitable for narrow flow field and high flow velocity. The model is coupled with the electric field, the flow field, and the temperature field to form a multi-physics field coupling model. In addition, dynamic simulation is carried out on account of the multi-physics field coupling model and comparative experiments are conducted using the self-developed ECM machine tool. While a shortcut appeared in the contrast experiment, machining with vibration-assisted channel ECM achieved fine machining stability and surface quality. The workpiece obtained by vibration-assisted channel ECM has three narrow and straight channels, with a width of less than 3 mm, an aspect ratio of more than 8, and an average surface roughness *Ra* in the hub of 0.327 μm. Compared with experimental data, the maximum relative errors of simulation are only 1.05% in channel width and 8.11% in machining current, which indicates that the multi-physics field coupling model is close to machining reality.

## 1. Introduction

Blisk is an important part of an advanced aeroengine [1]. However, owing to its extremely complex structure, difficult-machined material, and high manufacturing precision requirement, blisk machining has been an enormous challenge for the manufacturing industry [2]. As an advanced processing method removing materials in the form of ions, electrochemical machining (ECM) is accessible to achieve high surface quality, being free from the influence of the material hardness [3]. Therefore, ECM has become the main machining method of blisk [4]. Generally, blisk ECM is divided into two steps. That is, the cascade channel is pre-machined firstly in channel ECM and then the blisk surface is finished in profile ECM [5]. Due to the impact of memory error, a distribution of allowance before processing will seriously affect the profile ECM precision [6]. Thus, if the distribution of machining allowance after channel ECM is not uniform, it has a serious influence on the machining stability and accuracy in the following machining step [7]. Hence, blisk channel machining is a key machining process of blisk ECM.

Over the past few years, a considerable amount of research has been done in the blisk ECM process. Xu et al. proposed an efficient machining method of blisk channel ECM and presented an experimental system with the synchronous movement of three tool tubes, which can process multiple channels at the same time [8]. Zhu et al. proposed a method called rotating-cathode feeding electrochemical trepanning to homogenize the allowance distribution of twisted blades on a blisk in the roughing stage [9]. Wang et al. established a method of variable feed rate mode to process the cascade channel. This method could effectively reduce the difference of allowance in machining the blisk channel [10]. Klocke et al. constructed a simulation model of the ECM process for the engine blades and analyzed the changes of flow rate, gas volume fraction, temperature, and conductance in the ECM process [11]. Ernst et al. present an inverse approach for the tool optimization, and the tool shape for the desired vane geometry is calculated using a self-developed algorithm. The experimental validation of the simulated tool shape showed good results, especially for the forming of the flow surfaces and the leading edge [12].

Because of serious stray corrosion of non-machining surfaces in conventional linear or rotation feeding, it is difficult to get a complex curved surface with high precision. Numerous studies have focused on the application of electrode vibration in the ECM process to improve machining quality and stability. Hewidy et al. proposed a mathematical model for studying the mechanism of metal removal. The model shows that the hybrid ECM with low-frequency tool vibration has a positive effect on changing the physical conditions in the electrode gap [13]. Wang et al. processed a narrow slit by cathode compound feeding and proved that compound feeding can obtain a narrow slit with a more uniform average width [14]. Yue et al. showed that cathode vibration feed can improve the machining accuracy of micro-hole [15]. Bhattacharyya et al. studied the effect of the cathode vibration frequency on the machining accuracy of small holes [16]. Uhlmann et al. presented a new approach for modeling the development of surface roughness during vibratory finishing processes [17].

To reveal the coupling mechanism between the multiple physical fields and analyze the forming process of ECM, lots of researchers have established various theoretical models. For example, Klink et al. studied the formation of cavitation in ECM by investigating the electrolyte flow in narrow openings [18]. Zhao et al. optimized the hollow slice cathode structure and vibrating parameters by simulating the flow field [19]. Qu et al. proved that progressive pressure flow gives a high electrolyte flow rate through simulation, allowing a high cathode feed rate without a shortcut [20]. Tang et al. applied the electrolyte flow field mathematics models and the 3D gap flow field simulation geometric model to carry out the simulation research [21].

Blisk channel ECM has a narrow and twisted machining gap, in which parameters such as temperature, gas volume fraction, and electrolyte velocity change tremendously. Therefore, it is difficult to keep the process steady and obtain a high machining quality [22]. Vibration may be an effective way to improve machining stability and quality. So, this study adopted a cathode vibration feed ECM method for machining blisk channels. A cathode feeds towards the workpiece with a vibration movement of a cosine function curve, thereby improving the allowance uniformity of the machined blisk channel [23]. To analyze the influence of vibration on the forming process of ECM, the multi-physical field coupling model is established and the dynamic simulation is carried out based on the parameter transfer law of ECM. The effects of vibration ECM on electrolyte flow rate, bubble rate, conductivity, and stability were studied. The influence of each physical field on blisk channels ECM is analyzed, which provides theoretical guidance for experiments. Finally, experiments were carried out using the self-developed ECM machine tool to verify simulation results. To evaluate machining quality, the surface profile and workpiece geometrical size were measured.

## 2. Geometric Model of Blisk Channel Electrochemical Machining (ECM) 

**Figure 1:**
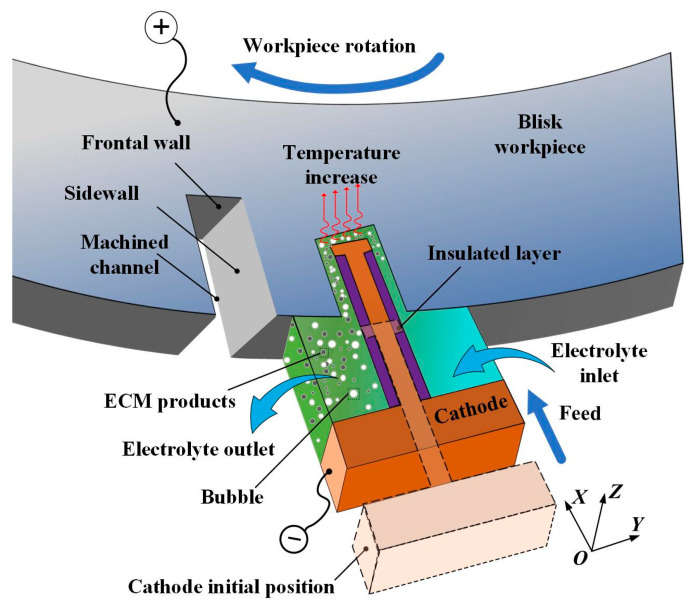
Principle of blisk channel electrochemical machining (ECM).

Figure 1 shows a schematic diagram of the blisk channel ECM process. During the channel ECM process, the workpiece is connected to the positive pole of the power supply and the cathode is connected to the negative pole of the power supply to form a closed circuit. Normally, a reduction reaction occurs on the surface of the cathode, and an oxidation reaction takes place on the surface of the workpiece. Electrolyte flows through the machining gap between the cathode and the workpiece at a high speed to take away the hydrogen, electrolytic products and remove the heat generated in the ECM process. After the channel machining is finished, the cathode returns to its initial position and the workpiece rotates for a certain angle to process the next channel.

## 3. Establishment of the Multi-Physical Coupling Model

### 3.1. The Interaction of Physical Fields in Electrochemical Machining (ECM)

**Figure 2:**
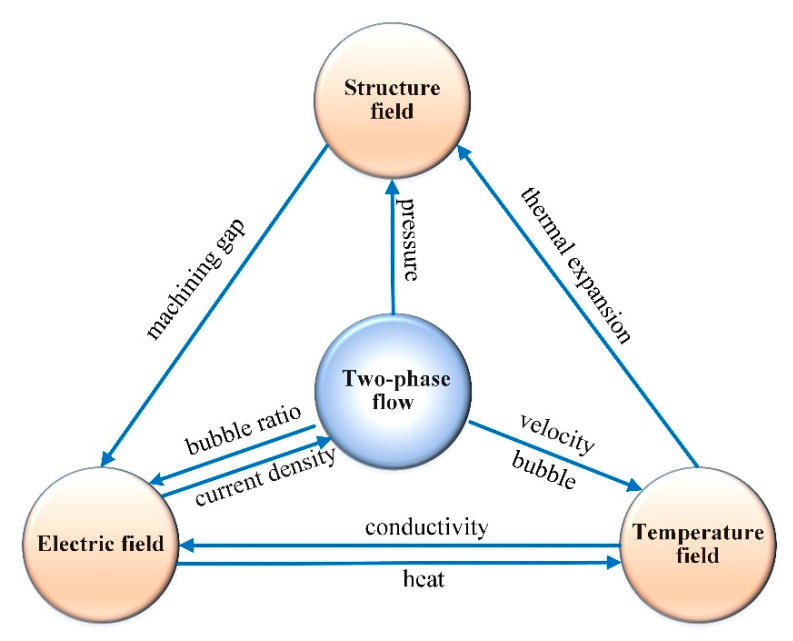
Multi-physical coupling relationship of ECM.

This paper studies the influence of cathode vibration feed on electrochemical machining. The coupling relations among the physical fields in channel ECM are shown in Figure 2. ECM is a complex machining process, including the electric field, the flow field, and the temperature field, these physical fields coupling each other and affecting the material removal on the workpiece. The change of a single physical field factor often affects the rest of the physics parameters and machining stability, resulting in the change of final machining stability and workpiece profiles. Therefore, it is of great significance to explore the dynamic forming process of ECM under coupling multi-physics.

### 3.2. Electric Field Modeling

To simplify electric field modeling, the following assumptions are made in the model:

(1) Electrolyte concentration polarization and electrochemical polarization are not considered in the ECM process, and the surface of the anode material is always assumed to be in an activated state [24].

(2) ECM process has entered an equilibrium state, which is only a function of position space and belongs to a steady-state current field [25].

(3) The machining products are uniformly corroded from the material surface, and the products have only a certain valence [26].

(4) The electrolyte used in ECM is isotropic and, therefore, the potential distribution of the electric field conforms to Laplace’s equation [27]. The model is established as follows:

(1) $$\frac{\partial^{2}\varphi }{\partial x^{2}}+\frac{\partial^{2}\varphi }{\partial y^{2}}=0$$

where *φ* is the potential in the inter-electrode gap, *x* and *y* are the coordinate values of each point in the model.

In the electric field simulation of ECM, the cathode is connected with the negative pole of the power supply and the anode is connected with the positive pole of the power supply. The potential of the anode surface is defined as *U* and the potential of the cathode surface is 0. Therefore, the first kind of boundary condition is satisfied in the electric field model [28]:

(2) $$\{\begin{array}{c} \varphi |\tau_{1}=0\\ \varphi |\tau_{8}=U \end{array}$$

For the other boundary, the second boundary condition is satisfied:

(3) $$\frac{\partial \varphi }{\partial n}|\tau_{2,3,4,5,6,7,9,10,11,12\;}=0\left(\mathrm{Insulation}\;\mathrm{boundary}\right)$$

The electric field intensity of each point in the processing zone is equal to the negative value of the potential gradient of the point [29]:

(4) $$\vec{E\;}=-\nabla \varphi$$

For the electrolyte in the processing area, the relation between the current density, the electric field strength, and conductivity is as follows [30]:

(5) $$\vec{i\;}=\kappa \vec{E\;}$$

where *i* is current density, *κ* is the electrolyte conductivity and *E* is electric field strength. The above formulas are the electric field mathematical model of ECM, which can be introduced into the Laplace equation to solve the electric field distribution at any point in the machining area. Combined with the Lagrange-Euler (ALE) method, the dynamic simulation under the action of the electric field can be completed.

### 3.3. Flow Field Modeling

In the process of ECM, hydrogen and hydroxide products are generated near the cathode surface and anode surface, respectively. Since it is reasonable to neglect the influence of the hydroxide products, a two-phase flow is adopted in the model. In the turbulent bubble flow module, it is assumed that the gas produced by electrolysis follows the ideal gas state equation and the current efficiency is approximately constant.

The flow field in the gap of the machining area is a typical gas-liquid two-phase flow. The continuous equation of liquid phase mass in the gap of electrochemical machining area is shown in Equation (6); the mass equation of hydrogen gas phase is shown in equation:

(6) $$\left(1-\beta \right){\Delta u=\Delta }_{0}u_{0}$$

(7) $$\frac{p}{R_{g}T}{\beta \Delta u=\eta }_{g}k_{g}ix$$

in which *β* is bubble rate, *p* is pressure, *η_g_* is current efficiency of hydrogen evolution, *k_g_* is hydrogen evolution mass electrochemical equivalent, and *R_g_* is hydrogen gas state parameter.

The influence of the bubble and temperature distributions on the electrolyte conductivity is expressed as follows [31]:

(8) $$\kappa \;{=\kappa }_{0}\left[1+\alpha \left(T_{k}-{\;T}_{0}\right)\right]{\left[1\;-\;\beta \right]}^{n}$$

in which *κ*_0_ is the initial electrolyte conductivity, *α* is the temperature coefficient of conductivity, *T_k_* is the electrolyte temperature, and *T*_0_ is the initial electrolyte temperature. *β* is the void fraction, *n* is the influence coefficient of bubble rate on conductivity, usually 1.5–2. On the one hand, the bubble rate increases in the ECM process, which leads to a decrease in electrolyte conductivity. On the other hand, the increase in electrolyte temperature will cause an increase in electrolyte conductivity.

According to Faraday’s law, the amount of hydrogen produced per unit of time and per unit of area on the surface of the cathode can be obtained [32]:

(9) $$\beta =\frac{Mi\eta }{2F}$$

where *M* is the molar mass of hydrogen, *i* is current density, *F* is Faraday’s constant, *F* = 96,500 [A∙s∙mol], and *η* is current efficiency.

According to the existing actual processing conditions, electrolyte velocity is *u* = 16 m/s, the hydraulic diameter is *D_h_* = 0.97 mm, and the kinematic viscosity of 10% sodium nitrate solution is *ν* = 0.80 × 10^−6^ m^2^/s under the condition of 30 °C. The Reynolds number is calculated by the equation:

(10) $$R_{e}=\frac{uD_{h}}{\nu }=1{.94\;\times \;10}^{4}$$

According to the Reynolds number, the electrolyte is in a turbulent state (*R_e_* >> 2300) [33]. Considering the influence of bubbles, the RANS *k-ε* turbulence model is suitable for the current situation and is adopted to simulate the flow field in this study [34].

(11) $$\frac{\partial_{k}}{\partial_{T}}+\nabla [k\vec{u}-(v+\frac{v_{T}}{\sigma_{k}})\nabla k]=P_{k}+S_{k}-\varepsilon$$

(12) $$\frac{\partial_{\varepsilon }}{\partial_{t}}+\nabla [\varepsilon \vec{u}-(v+\frac{v_{T}}{\sigma_{\varepsilon }})\nabla \varepsilon ]=\frac{\varepsilon }{k}(C_{1}P_{k}+C_{\varepsilon }S_{k}-C_{2}\varepsilon )$$

(13) $$P_{k}=\frac{v_{T}}{2}{|\nabla_{\vec{u}}+{\left(\nabla_{\vec{u}}\right)}^{T}|}^{2}$$

(14) $$S_{k}=-\beta C_{k}{\left|\nabla_{p}\right|}^{2}$$

where *k* is the turbulent energy, *ε* is the turbulent dissipation, *P_k_* is the generating term of turbulent energy, *σ_k_* and *σ_ε_* are Prandtl numbers corresponding to “*k*” and “*ε*”, *u* is the flow velocity, and *C*_1_, *C*_2_, *C_ε_*, *C_k_*, *δ_ε_* is the model constant.

### 3.4. Temperature Field Modeling

The temperature distribution in the machining gap can be calculated by the convection–diffusion equation [35]:

(15) $$d_{z}\rho C_{P}\frac{\partial T}{\partial t}+d_{z}\rho C_{P}u\nabla T+d_{z}\nabla (-\lambda \nabla T)=d_{z}H+q_{0}$$

where *d_z_* is the thickness of the boundary layer, *C_p_* is the electrolyte-specific heat capacity, *λ* is the electrolyte thermal conductivity, *q*_0_ is initial heat flux, *u* is velocity field of the liquid phase of electrolyte, *ρ* is the density of the electrolyte, and *H* is the heat source, the Joule heat generated in the flow channel.

The Joule heat *H* generated in the flow channel is as follows [36]:

(16) H=E×j

where *E* is electric field strength*, j* is the local current density.

The convective heat flux at the surface boundary of cathode and anode is as follows:

(17) $$q_{1}=h\times (T_{\mathrm{ext}}-T_{k})$$

where *q*_1_ is convective heat flux, *h* is heat transfer coefficient and *T_ext_* is external temperature.

### 3.5. Deformation Field

There are two main feeding methods in ECM: linear feed and cathode vibration feed. In the linear feed mode, the cathode moves in a straight line at a constant speed and its feed trajectory is linear, which can be expressed by the equation:

(18) $$s=B_{0}+V_{1}\times t$$

The cathode vibration feed makes a periodic vibration with a certain amplitude while making a linear feed motion, and its trajectory is a sinusoidal motion of continuous feed, which can be expressed by the equation:

(19) $$s=B_{0}+V_{1}\times t-A_{0}\cos wt$$

where *s* is cathode position, *B*_0_ is initial machining clearance, *V*_1_ is the cathode feed rate, *t* is the machining time, *A*_0_ is the amplitude of the displacement of the tool cathode, and *ω* is angular velocity.

**Figure 3:**
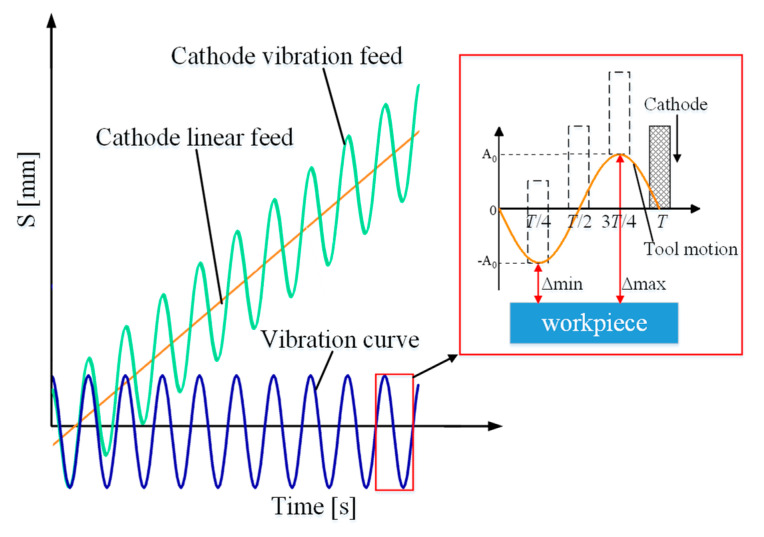
The law of cathode movement.

Figure 3 is a comparison of the cathode movement law in the two feed modes. The cathode vibration feed mode is developed from the linear feed method, with an auxiliary back and forth movement. When the vibration frequency is 0, it is the linear feed mode.

## 4. Simulation Analysis

### 4.1. Model Description

It is shown in Figure 1 that the cross-sectional shape is approximately the same when its normal direction of the section is *Z*. Therefore, a two-dimensional cross-sectional shape is employed to simplify the research problem, thus reducing the amount of calculation in the simulation process and improving the simulation efficiency.

**Figure 4:**
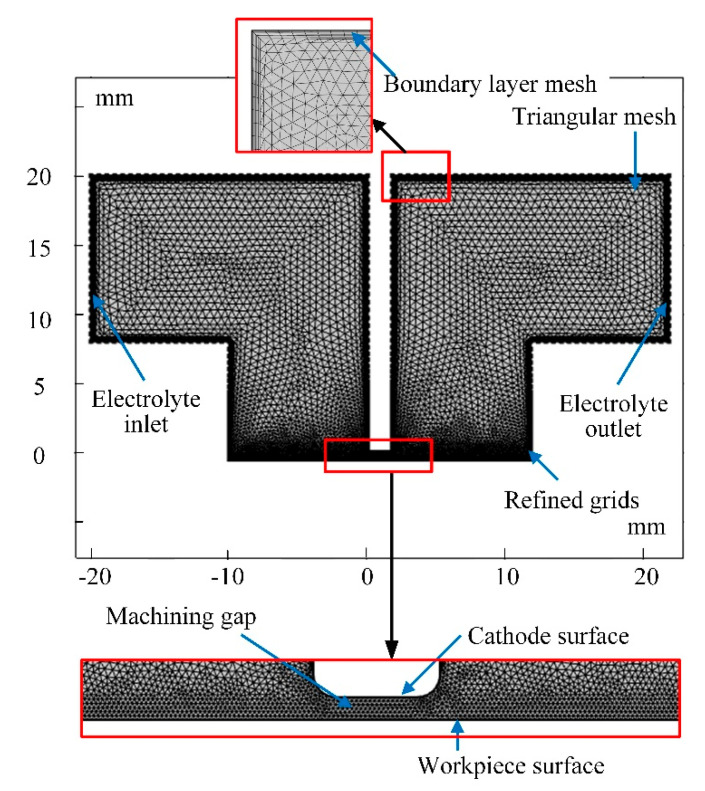
Mesh generation of the model.

Based on the above multi-field coupling model, COMSOL Multiphysics 5.4 is used to carry out a multi-physics simulation of the ECM process of the blisk channel. The 2D model and mesh division of the simulation is shown in Figure 4. Since the dynamic simulation will lead to a large deformation on the anode surface, the partial mesh at the anode boundary area with large deformation is refined locally to ensure the accuracy of the simulation results.

### 4.2. Parameters Setting

The relevant parameters should be set before the simulation analysis. The simulation parameters of the multi-physical field coupling model are consistent with the actual machining. The process parameters are shown in Table 1.

### 4.3. Boundary Condition

**Figure 5:**
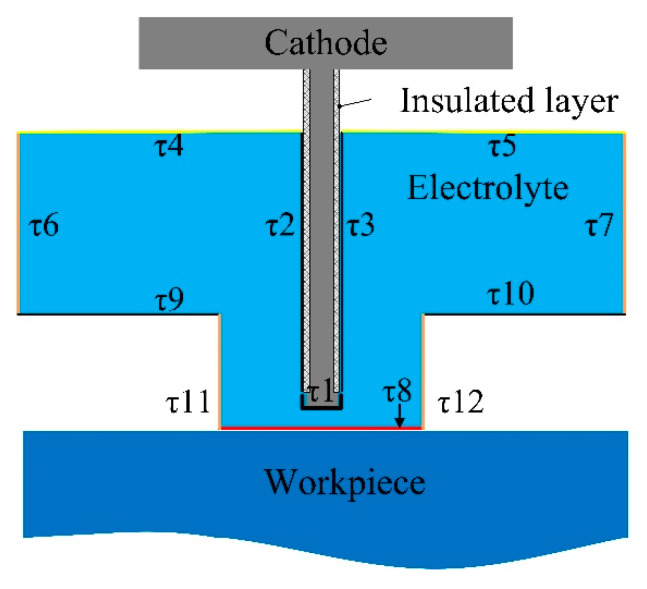
Boundary distribution in simulation.

The dynamic simulation under the action of ECM multi-physical field coupling is carried out by using COMSOL software’s four modules: primary current distribution, deformation geometry, bubble flow *k-ε* (RANS *k-ε*), and fluid heat transfer. The simulation model of ECM multi-physical field coupling is shown in Figure 5 and the boundary conditions are shown in Table 2.

### 4.4. Simulation Results and Discussion

The cathode vibrates according to the cosine curve and the machining gap changes periodically. In a single vibration cycle, three cathode positions under the balance state of vibrational feed ECM are selected, which are the nearest cathode position to the anode (*t* = *T*/4), cathode at the middle position (*t* = *T*/2), and cathode at its farthest position to the anode (*t* = 3*T*/4).

**Figure 6:**
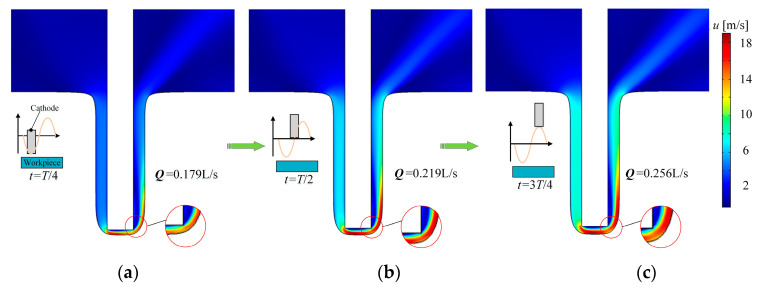
Distributions of electrolyte velocity over time; (**a**) *t* = *T*/4. The nearest cathode position to the anode, the maximum flow is 0.179 L/s; (**b**) *t* = *T*/2. Cathode at the middle position, the maximum flow increases to 0.219 L/s; (**c**) *t* = 3*T*/4. Cathode at its farthest position to the anode, the maximum flow reaches 0.256 L/s.

Figure 6 shows the velocity distributions at different times in the ECM simulation. In the maps of velocity distribution at different times, it can be seen intuitively that the flow of electrolyte in the machining area is 0.179 L/s at *T*/4. With the retreat of the cathode caused by vibrational motion, the maximum flow reaches 0.219 L/s at *T*/2 and 0.256 L/s at 3*T*/4. Therefore, the renewal of the electrolyte and the exhaustion of the electrolysis products are improved by the increase of electrolyte flow velocity. The improvement in the flow field is beneficial to reinforce the machining stability and accuracy of blisk channels machining.

**Figure 7:**
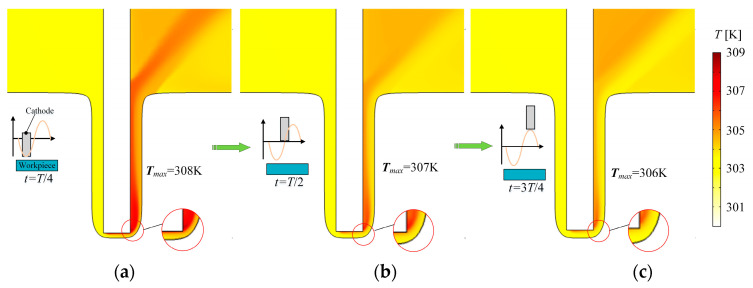
Distributions of electrolyte temperature over time; (**a**) *t* = *T*/4. The maximum temperature is 308 K at the cathode’s nearest position to anode; (**b**) *t* = *T*/2. The maximum temperature drops to 307 K at the middle position; (**c**) *t* = 3*T*/4. The maximum temperature is 306 K at the farthest position.

The temperature distributions in the machining area in a unit cycle are obtained through transient simulation, as shown in Figure 7. The heat generated in the ECM process gradually accumulates and the temperature in the machining gap gradually increases along the flow direction. Due to the small machining gap and increasing machining depth, the heat is difficult to eliminate. The temperature is 303 K in the inlet wall and increases to 308 K to the side of the outlet at *T*/4, as shown in Figure 7a. Figure 7b,c indicate that when the cathode moves far away from the workpiece, the machining gap becomes larger, and therefore, the flushing electrolyte quickly takes away the heat and reactants.

**Figure 8:**
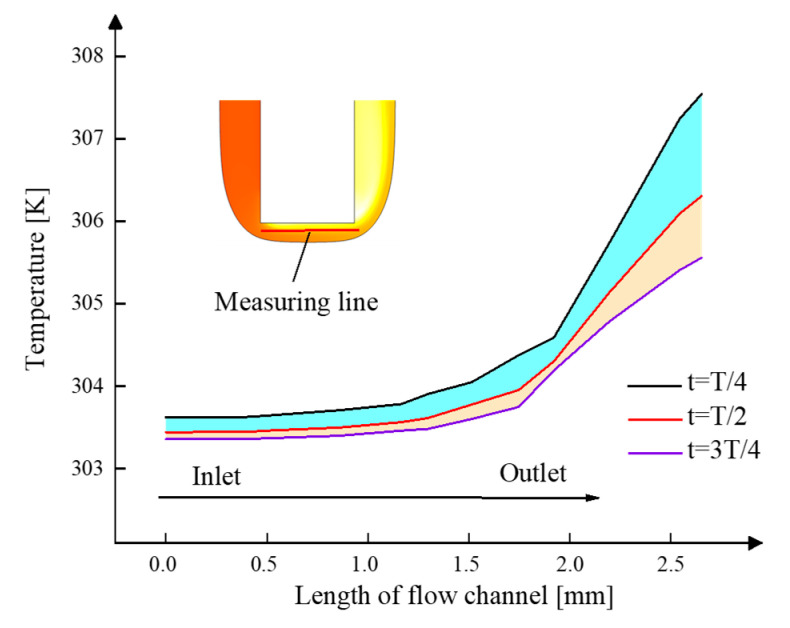
Temperature distribution of electrolyte near the cathode.

To study the temperature evolution more exactly, the line between the cathode and the anode is used as a reference line. We plotted the curves of temperature along this line in Figure 8. It is observed that the temperature near the inlet is much lower than that near the outlet. Thus, the temperature in the channel increases along the direction of electrolyte flow. The maximum temperature appears on the outlet side and its value is 307.5 K at *T*/4. The temperature in the whole line has an evident drop at *T*/2 and 3*T*/4. The maximum temperature in this line drops to 305.5 K and it still appears at the outlet side at 3*T*/4. This is consistent with the result shown in Figure 7.

**Figure 9:**
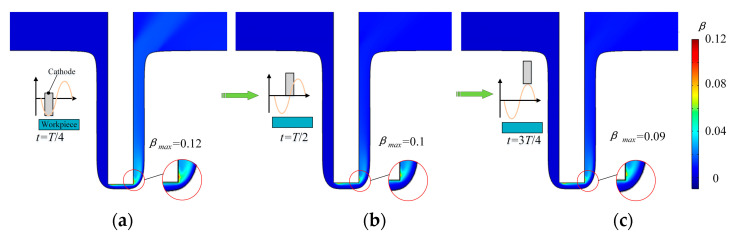
Distributions of gas-phase volume fraction over time; (**a**) *t* = *T*/4. The maximum volume fraction of hydrogen bubble is 0.12 at the cathode’s nearest position to anode; (**b**) *t* = *T*/2. The maximum volume fraction of the hydrogen bubble is 0.1 at the middle position; (**c**) *t* = 3*T*/4. The maximum volume fraction of the hydrogen bubble is 0.1 at the cathode’s farthest position.

The evolution of gas-phase volume fraction in the machining area in a unit cycle is obtained through a transient simulation shown in Figure 9. The volume fraction of gas in the whole gap increases over time and its content becomes higher along the flow direction, as shown in Figure 9a. The volume fraction of hydrogen bubble rises to 0.12 at *T*/4. In comparison, when the cathode moves away from the anode, it drops dramatically. As shown in Figure 9b,c, when the cathode moves away from the anode, the maximum value of the bubble volume fraction drops to 0.09. Therefore, the gas volume fraction drops greatly and thus the flow field of electrolyte in the inter-electrode gap is optimized obviously.

**Figure 10:**
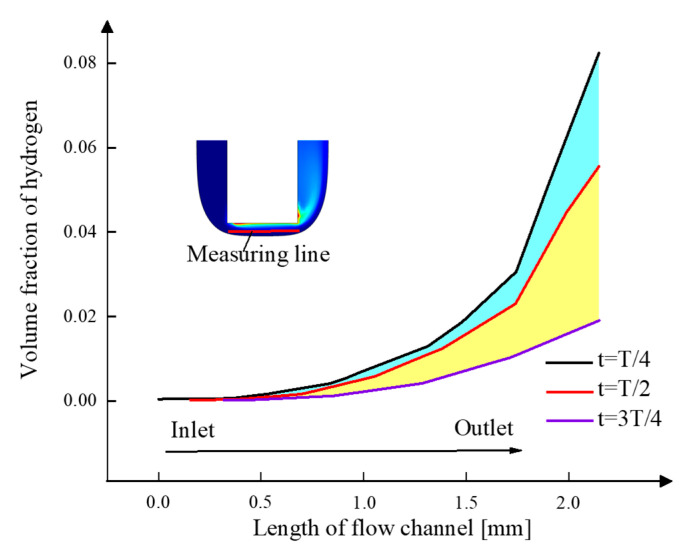
The volume fraction of hydrogen distribution of electrolyte.

Figure 10 presents the curves of electrolyte volume fraction along the flow direction during one period. The volume fraction of hydrogen increases along the direction of electrolyte flow in the machining area. The vibration of the tool cathode has a significant influence on the diffusion of gas bubbles. As the cathode moves away from the workpiece, the gas volume fraction starts to drop. The maximum gas fraction drops from 8.2% at *T*/4 to 1.9% at 3*T*/4. The drop of the gas fraction is of great benefit to improve the machining stability and quality.

## 5. Experiment Analysis

### 5.1. Experimental Design

**Figure 11:**
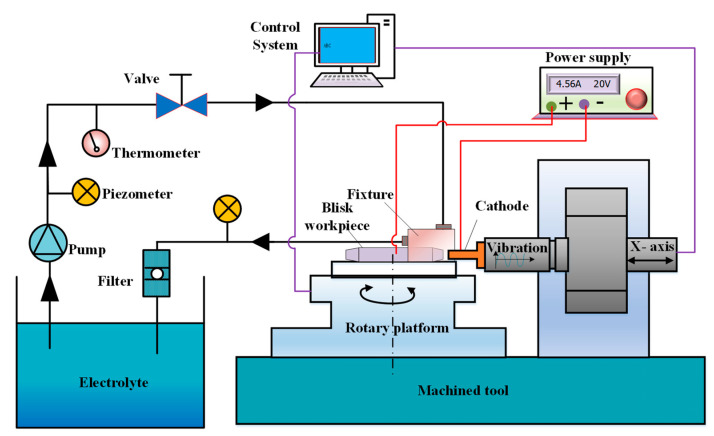
The set-up for electrochemical machining.

**Figure 12:**
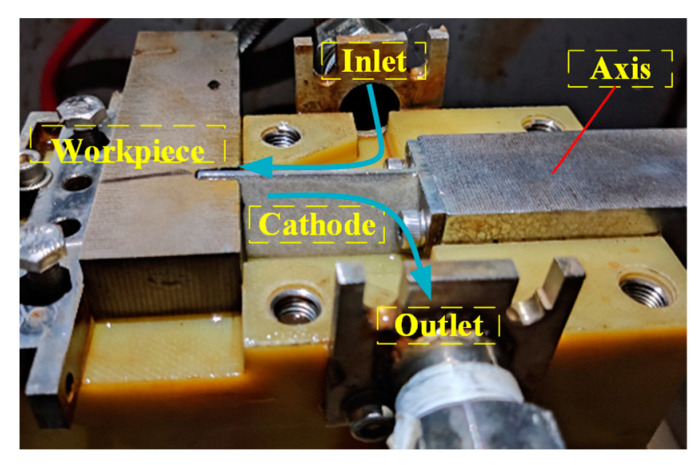
Electrochemical machining fixture.

To verify the simulation results, machining experiments are conducted with an ECM machine tool, as shown in Figure 11. This system comprises the rotary platform, feed and vibration system, power supply, electrolyte circulation and filtration system, processing fixture, and integrated control system. The processing fixture and its flow field are shown in Figure 12. The ECM fixture provides a sealed flow channel with the cathode and workpiece. The electrolyte flow form is a side flow type, in which the electrolyte inlet and the electrolyte outlet are at the different sides of the fixture. The high-speed electrolyte flows into the machining area from the electrolyte inlet pipe, passes through the inter-electrode gap, and returns to the electrolyte circulation system for filtering and recycling.

In the experiments, samples of Inconel 718 superalloy are chosen as the workpiece (anode). The electrolyte is 10% NaNO_3_ and its temperature is 30 °C. The electrolyte is pumped through the channel at a high speed by the constant pressure pump and the electrolyte pressure at the inlet is 0.3 MPa. The power supply is 22 V. To investigate the influence of the cathode feed mode on the machining stability, contrast experiments using a cathode linear feed are also performed. Workpieces have the same feed velocity of 0.5 mm/min in the two modes. After machining, the width of the machined channel is measured along the depth direction.

### 5.2. Analysis of Experimental Results

**Figure 13:**
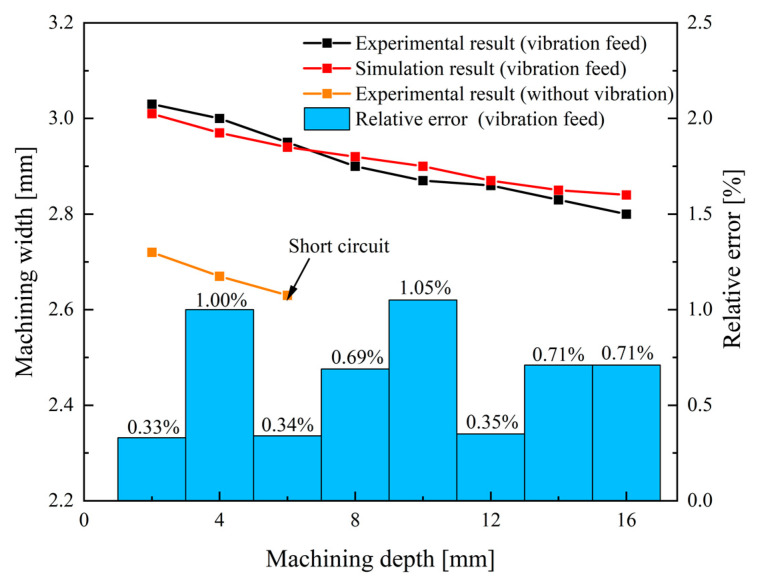
Variation of the channel width with different feed modes.

**Figure 14:**
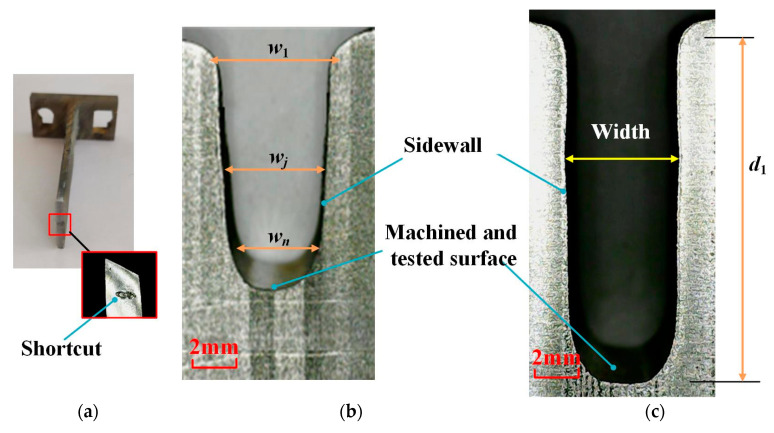
Morphology of blisk channels under different feed modes; (**a**) shortcut region of the cathode; (**b**) workpiece obtained in contrast experiment; (**c**) workpiece obtained by the vibration-assisted channel ECM.

Figure 13 shows the variation of the channel width in different feed modes. The average width of the blisk channel processed through vibration-assisted channel ECM is slightly wider than that processed through the linear feed ECM without vibration. Figure 14 shows the morphologies of the channel in the two types of feed modes.

A short circuit occurs between the anode and cathode when the machined depth is 7 mm in the contrast experiment. The shortcut region is located at the front of the cathode, shown in Figure 14a. Since electrolytic products and heat are generated in the machining process, it requires high-speed flowing electrolyte to take them away from the machining area. As machining depth grows, the narrow flow channel becomes longer and the pressure loss in the flow passage increases. As a result, when the electrolyte flow speed decreases more and more dramatically, products and heat cannot be completely removed from the machining area, affecting the stability of machining.

In the vibration-assisted channel ECM experiment, the sidewalls are close to straight lines and the width of the channel has no obvious change, as shown in Figure 14c. The vibration produces a strong suction effect in the machining gap. Moreover, when the cathode goes back, it is helpful to update the electrolyte and accelerate the heat exchange, which can effectively avoid the occurrence of a short circuit phenomenon. Therefore, compared with the linear feed mode, the machining stability and the machining quality of the ECM of the blisk channel are improved in the vibration feed mode.

In this paper, sidewall taper *θ* of the channel is defined as follows [37]:

(20) $$\mathrm{Taper}(\theta )=\frac{\mathrm{atan}\left(w_{1}-w_{n}\right)}{2d_{1}}$$

where *d*_1_ is the depth of the machined channel, *w*_1_ is the width of a machined channel on the outside position, and *w_n_* is the width of a machined channel on the inside position. The sidewall taper of the blisk channel processed through the cathode linear feed is 0.86°. When the cathode was fed by vibration, the width of a channel was enlarged, while the taper was 0.82° and the change of taper is not obvious.

The effects of the cathode feed mode on the machining accuracy are investigated by analyzing the average of the machined channel width $\bar{w}$, the standard deviation width of the machined channel *σ_a_* and *σ_b_*. The average machined channel width $\bar{w}$ is defined as follows:

(21) $$\bar{w}=\frac{w_{1}+w_{2}+\ldots w_{n}}{n}$$

The standard deviation of the machined channel width *σ*_1_ and *σ*_2_ are defined as follows:

(22) $$\sigma_{a}=\sqrt{\frac{\sum_{\Sigma =1}^{n}{\left(w_{j}-\bar{w}\right)}^{2}}{n}}$$

(23) $$\sigma_{b}=\sqrt{\frac{\sum_{\Sigma =1}^{n}{\left(w_{j}-w_{\theta j}\right)}^{2}}{n}}$$

(24) $$w_{\theta j}=w_{1}-2\times D_{1j}\times \tan\theta$$

where *w_j_* is the width of a machined channel in the *j* position, *w_θj_* is the width of a machined channel in the *j* position on the taper line, and *D*_1*j*_ is the distance between position 1 and position *j*. The standard deviation of the machined channel width *σ_a_* reflects the straightness of the sidewall, which is affected by the taper to some extent. The standard deviation width *σ_b_* eliminates the influence of taper. Table 3 demonstrates the average of the machined channel width and the standard deviation width with different feed modes. The channel obtained by the vibration-assisted channel ECM method was wider than that obtained in the contrast experiment. Since a short circuit appeared in the contrast experiment, the depth of the channel obtained in the contrast experiment was shorter than the other one. For that, the value of *σ_a_* (=0.0076 mm) is much higher than *σ_b_* (=0.0027 mm), thus taper of the channel has an obvious influence on the channel profile. The value of *σ_b_* is only 0.0027 mm in the whole channel (with a depth of 16 mm), which also indicates that the process is very stable.

**Figure 15:**
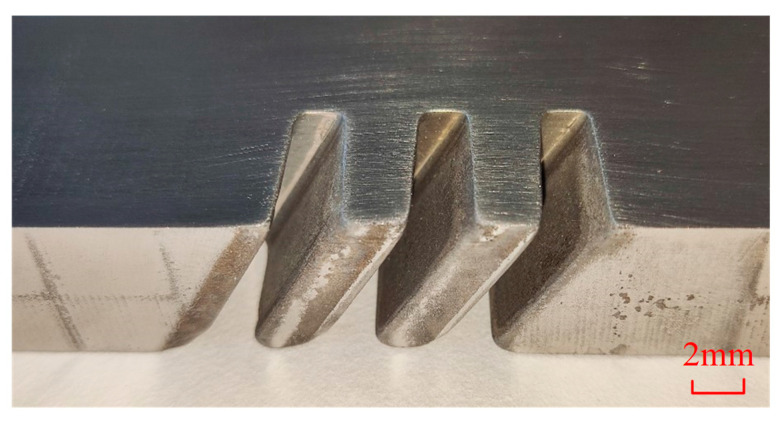
Machined specimen with the channels.

**Figure 16:**
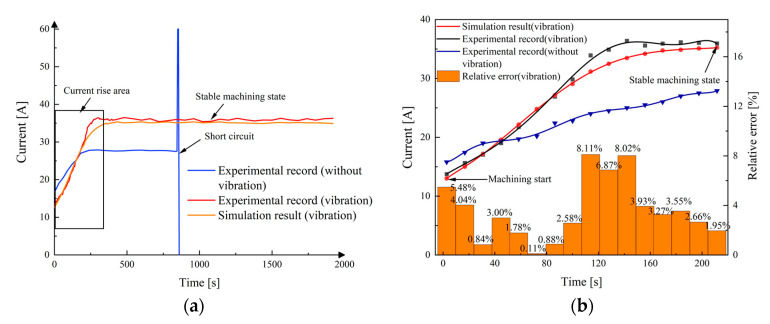
The changes of machining current experiment and simulation over time; (**a**) whole machining time; (**b**) enlarged view of current in rising time.

Figure 15 demonstrates the machined workpiece with vibration-assisted channel ECM. The workpiece obtained by the cathode vibration feed method has three narrow channels, with a width of less than 3 mm and an aspect ratio of more than 8. Figure 16 shows the changes of current in the machining area recorded in the experiment and simulation over time. The current in channel machining rose from 13.01 A at the beginning to 35.94 A at 211 s (current rising period) and then kept almost constant (stable machining state). The current achieved a stable state during processing in the balance period, which indicates the process is reliable. It was obvious that the trend of change of experimental current and simulation current density is roughly the same, with the maximum relative error of only 8.11%, as shown in Figure 16.

**Figure 17:**
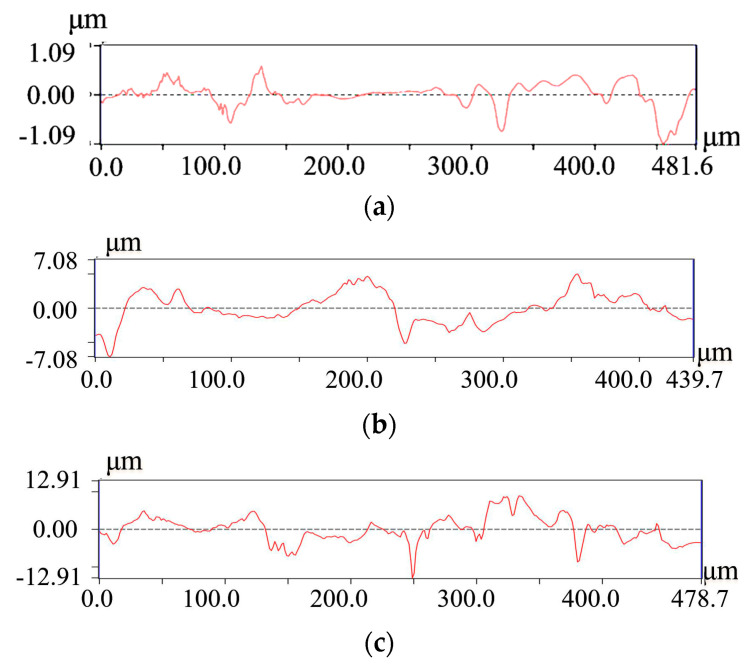
The profile curve of Inconel 718 the channel machining; (**a**) the channel hub; (**b**) the sidewall close to the inlet; (**c**) the sidewall close to the outlet.

**Figure 18:**
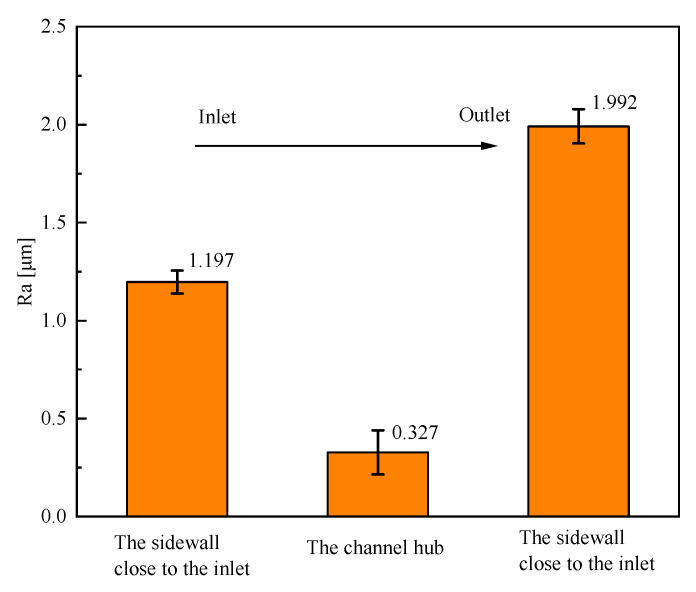
Surface roughness *Ra* of the channels.

To evaluate the surface quality, the JD-520 roughness tester was used to measure the roughness of the hub, electrolyte inlet wall, and outlet wall. The machined surfaces of the specimens were measured using the roughness tester. Then, the surface profile curves were obtained, as shown in Figure 17. The values of roughness *Ra* are listed in Figure 18. The average surface roughness *Ra* in the hub, the sidewall close to the inlet, and the sidewall close to the outlet are 0.327 μm, 1.197 μm, and 1.992 μm, respectively. The value of *Ra* at the hub is lower than that at the sidewalls. One of the reasons is that the electrolyte flow rate near the hub is higher than that near the electrolyte inlet and outlet. The other reason is that secondary corrosion occurs at the machined sidewall, resulting in the decreases of the surface roughness and surface quality of the sidewall. At the same time, it is observed that the value of *Ra* in the sidewall close to the inlet is less than the sidewall close to the outlet. The reason for the different machining quality is that when most products and bubbles are generated in the hub of the blisk channel, the electrolyte carries the electrolytic products to the outlet, resulting in uneven changes in conductivity and affecting the surface quality.

**Figure 19:**
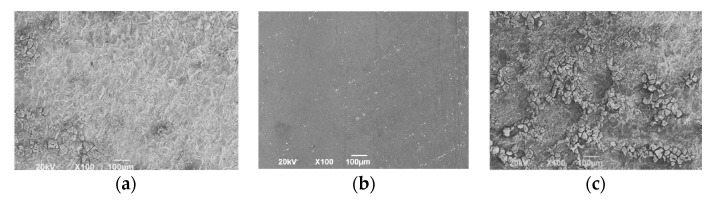
Surface morphology of the machined Inconel 718 channel; (**a**) the sidewall close to the inlet; (**b**) the channel hub; (**c**) the sidewall close to the outlet.

The surface topographies of the channel hub, the electrolyte inlet wall, and the outlet wall were scanned by scanning electron microscope (SEM), shown in Figure 19. The surface of the hub is much smoother. There is a certain degree of uneven corrosion on the sidewalls of the channel, presenting block discrete distribution morphology. In addition, the uneven corrosion degree of the sidewall close to the outline is higher than that of the sidewall close to the inlet. Therefore, the surface quality of the sidewall at the electrolyte inlet is slightly better than that at the outlet, which is consistent with the roughness data measured in Figure 18.

## 6. Conclusions

In the present research, a theoretical model of multi-field coupling is established to analyze the influence of vibration on the blisk channel ECM. Based on the multi-physical field simulation results and experimental investigations, the conclusions are summarized as follows:

(1) A multi-physical field coupling model, including temperature field, flow field, electric field, is established for the blisk channel ECM based on the transmission relationship of machining parameters. The model is used to obtain the spatial distribution of temperature, hydrogen volume fraction, electrolyte conductivity, and other parameters.
(2) Simulations show that vibration-assisted channel ECM can effectively promote the exclusion of electrolytic products and the renewal of the electrolyte in the machining gap. As the cathode moves away from the workpiece, the gas volume fraction and electrolyte temperature have an evident drop. The drops of gas fraction and electrolyte temperature are of great benefit to improve the machining stability and quality.
(3) The variation trend of the workpiece contour shape obtained by the experiment and simulation is consistent. Comparing the channel width between simulation and experiment, the maximum relative error is only 1.05%. A comparison of the currents between the simulation and experiment was also conducted, with the maximum relative error of only 8.11%.
(4) An Inconel 718 alloy blisk with three narrow channels has been successfully manufactured by vibration-assisted channel ECM. The experimental results show that the cathode vibration significantly improves the machining stability and surface quality. The width of the narrow channels is less than 3 mm and the aspect ratio is more than 8. The average surface roughnesses *Ra* of the hub is 0.327 μm.

## Figures and Tables

**Table 1:** Simulation parameters.

Symbol	Definition	Value	Unit
***T*** **_0_**	Initial temperature	303.15	K
***P*** **_0_**	Inlet pressure	0.3	MP_a_
***P*** **_1_**	Outlet pressure	0	MP_a_
***V*** **_0_**	Feed rate	0.5	mm/min
***k*** **_0_**	Electrolyte conductivity	7.6	S·m
***U***	Processing voltage	22	V
***f***	Frequency of oscillation and pulse	5	Hz
***A***	Amplitude	0.2	mm

**Table 2:** Boundary conditions setting of multiple physical fields.

	Boundary	Condition Setting
Flow Field	*τ*1	The gas flux *V_H_* = (*H*_2_*·cd.IlMag*)/(2·*F*)
	*τ*2, *τ*3, *τ*4, *τ*5, *τ*8, *τ*9, *τ*10, *τ*11, *τ*12	No sliding wall and no gas flux at the boundary
	*τ*6	Electrolyte inlet, pressure boundary (0.3 MPa)
	*τ*7	Electrolyte outlet, pressure boundary (0 MPa)
Temperature Field	*M*	Heat source, total power density
	*τ*1, *τ*8	Boundary heat source of electrochemical reaction
	*τ*2, *τ*3, *τ*4, *τ*5, *τ*9, *τ*10, *τ*11, *τ*12	Natural convection heat flux
	*τ*6, *τ*7	Temperature boundary, 303.15 K
Electric Field	*τ*1	Cathode surface, *U* = 0
	*τ*8	Anode surface, *U* = 20 V
	*τ*2, *τ*3, *τ*4, *τ*5, *τ*6, *τ*7, *τ*9, *τ*10,*τ*11, *τ*12	$\frac{\partial \varphi }{\partial n}|\tau_{2,3,4,5,6,7,9,10,11,12}=0$ (Insulation boundary)
	*M*	$\kappa =\kappa_{0}\left[1+\alpha (T_{k}-T_{0})\right]{\left[1-\beta \right]}^{n}$

**Table 3:** Evaluation of the machined channel width.

	Experimental Result (Vibration)	Simulation Result (Vibration)	Experimental Result (without Vibration)
*θ* (°)	0.82°	0.61°	0.86°
$\bar{w}$ (mm)	2.905	2.912	2.673
*σ*_a_ (mm)	0.076	0.056	0.037
*σ*_b_ (mm)	0.027	0.024	0.012

## References

[1] Bewlay B.P., Weimer M., Kelly T., Suzuki A., Subramanian P.R. (2013). The science, technology, and implementation of TiAl alloys in commercial aircraft engines. Mater. Res. Soc. Symp. Proc..

[2] Li X.Y., Ren J.X., Tang K., Zhou Y.K. (2019). A Tracking-based numerical algorithm for efficiently constructing the feasible space of tool axis of a conical ball-end cutter in five-axis machining. Comput. Aided Des..

[3] Ren Z., Wang D., Cui G., Cao W., Zhu D. (2021). Optimize the flow field during counter-rotating electrochemical machining of grid structures through an auxiliary internal fluid flow pattern. Precis. Eng..

[4] El-Hofy H. (2019). Vibration-assisted electrochemical machining: A review. Int. J. Adv. Manuf. Technol..

[5] Hu X.Y., Zhu D., Li J.B., Gu Z.Z. (2019). Flow field research on electrochemical machining with gas film insulation. J. Mater. Process. Technol..

[6] Zhang J.C., Zhu D., Xu Z.Y., Zhang K.L., Liu J., Qu N.S. (2016). Improvement of trailing edge accuracy in blisk electrochemical machining by optimizing the electric field with an extended cathode. J. Mater. Process. Technol..

[7] Zong Y.W., Liu J., Zhu D. (2021). Study of voltage regulation strategy in electrochemical machining of blisk channels using tube electrodes. Int. J. Adv. Manuf. Technol..

[8] Xu Z.Y., Xu Q., Zhu D., Gong T. (2013). A high efficiency electrochemical machining method of blisk channels. CIRP Ann..

[9] Lei G.P., Zhu D., Zhu D. (2021). Feeding strategy optimization for a blisk with twisted blades in electrochemical trepanning. J. Manuf. Process..

[10] Wang J., Xu Z.Y., Wang J.T., Zhu D. (2021). Electrochemical machining on blisk channels with a variable feed rate mode. Chin. J. Aeronaut..

[11] Klocke F., Zeis M., Klink A. (2015). Interdisciplinary modelling of the electrochemical machining process for engine blades. CIRP Ann.-Manuf. Technol..

[12] Ernst A., Heib T., Hall T., Schmidt G., Bähre D. (2018). Simulation of the tool shape design for the electrochemical machining of jet engine vanes. Proc. CIRP.

[13] Hewidy M., Ebeid S., El-Taweel T., Youssef A. (2007). Modelling the performance of ECM assisted by low frequency vibrations. J. Mater. Process. Technol..

[14] Wang F., Zhao J.S., Zhang X.L., Yang Z.W., Gan W.M., Tian Z.J. (2017). Electrochemical machining of a narrow slit by cathodic compound feeding. Int. J. Adv. Manuf. Technol..

[15] Pan Y., Xu L.Z. (2015). Vibration analysis and experiments on electrochemical micro-machining using cathode vibration feed system. Int. J. Precis. Eng. Manuf..

[16] Bhattacharyya B., Malapati M., Munda J., Sarkar A. (2007). Influence of tool vibration on machining performance in electrochemical micro-machining of copper. Int. J. Mach. Tools Manuf..

[17] Uhlmann E., Dethlefs A., Eulitz A. (2014). Investigation into a geometry-based model for surface roughness prediction in vibratory finishing processes. Int. J. Adv. Manuf. Technol..

[18] Klink A., Heidemanns L., Rommes B. (2020). Study of the electrolyte flow at narrow openings during electrochemical machining. CIRP Ann..

[19] Zhao J.S., Lv Y.M., Wang F., Yang Z.W., Liu D.M., Fan Y.T. (2018). Experimental research on process stability in pulsed electrochemical machining of deep narrow grooves with high length-width ratio. Int. J. Adv. Manuf. Technol..

[20] Qu N.S., Hu Y., Zhu D., Xu Z.Y. (2014). Electrochemical machining of blisk channels with progressive-pressure electrolyte flow. Mater. Manuf. Process..

[21] Tang L., Zhu Q.L., Zhao J.S., Fan Z.J. (2016). Research on the cathode design and experiments of electrochemical machining a closed impeller internal flow channel. Int. J. Adv. Manuf. Technol..

[22] Wang J., Xu Z.Y., Wang J.T., Xu Z.L., Zhu D. (2021). Electrochemical machining of blisk channels with rotations of the cathode and the workpiece. Int. J. Mech. Sci..

[23] Wang F., Zhao J.S., Lv Y.M., Yang Z.W., Yao J., He Y.F. (2017). Electrochemical machining of deep narrow slits on TB6 titanium alloys. Int. J. Adv. Manuf. Technol..

[24] Shenoy R.V., Datta M., Romankiw L.T. (1996). Investigation of island formation during through-mask electrochemical micromachining. J. Electrochem. Soc..

[25] Ghoshal B., Bhattacharyya B. (2013). Influence of vibration on micro-tool fabrication by electrochemical machining. Int. J. Mach. Tools Manuf..

[26] Toshiaki F., Kazuaki I., Kato D., Makoto Y. (2008). Multiphysics simulation of electrochemical machining process for three-dimensional compressor blade. J. Fluids Eng..

[27] Qian S.Q., Ji F., Qu N.S., Li H.S. (2014). Improving the localization of surface texture by electrochemical machining with auxiliary anode. Mater. Manuf. Process..

[28] Liu G., Tong H., Li Y., Zhong H. (2021). Novel structure of a sidewall-insulated hollow electrode for micro electrochemical machining. Precis. Eng..

[29] Collett D., Hewson-Browne R., Windle D. (1970). A complex variable approach to electrochemical machining problems. J. Eng. Math..

[30] Li D.L., Zhu D., Li H.S. (2011). Microstructure of electrochemical micromachining using inert metal mask. Int. J. Adv. Manuf. Technol..

[31] Deconinck D., Hoogsteen W., Deconinck J. (2013). A temperature dependent multi-ion model for time accurate numerical simulation of the electrochemical machining process. Part III: Experimental validation. Electrochim. Acta.

[32] Jiang X.C., Liu J., Zhu D., Wang M.M., Qu N.S. (2018). Research on stagger coupling mode of pulse duration and tool vibration in electrochemical machining. Appl. Sci..

[33] Zhu D., Zhang J.C., Zhang K.L., Liu J., Chen Z., Qu N.S. (2015). Electrochemical machining on blisk cascade passage with dynamic additional electrolyte flow. Int. J. Adv. Manuf. Technol..

[34] Li Z.L., Cao B.R., Dai Y. (2021). Research on multi-physics coupling simulation for the pulse electrochemical machining of holes with tube electrodes. Micromachines.

[35] Damme S., Nelissen G., Bossche B., Deconinck J. (2005). Numerical model for predicting the efficiency behaviour during pulsed electrochemical machining of steel in NaNO_3_. J. Appl. Electrochem..

[36] Deconinck D., Van D.S., Albu C., Hotoiu L., Deconinck J. (2011). Study of the effects of heat removal on the copying accuracy of the electrochemical machining process. Electrochim. Acta..

[37] Chen X.L., Zhu J.J., Xu Z.Z., Su G.K. (2021). Modeling and experimental research on the evolution process of micro through-slit array generated with masked jet electrochemical machining. J. Mater. Process. Technol..

